# Predictive factors for poor mobilization in autologous stem cell transplant: a multivariate model

**DOI:** 10.1016/j.htct.2026.106475

**Published:** 2026-05-30

**Authors:** Maryam Bagherian, Mahdi Jalili, Saeed Mohammadi, Hossein Kamranzadeh, Syed Asadollah Mousavi, Ahmadreza Shamshiri, Mohammad Vaezi, Shirin Ferdowsi

**Affiliations:** a Research Institute for oncology, Hematology and Cell Therapy, Tehran University of Medical Sciences, Tehran, Iran; b Cell Therapy and Hematopoietic Stem cell Transplantation Research Center, Research Institute for oncology, Hematology and Cell Therapy, Tehran University of Medical Sciences, Tehran, Iran; c Hematologic Malignancies Research Center, Research Institute for Oncology, Hematology and Cell Therapy, Tehran University of Medical Sciences, Iran; d Department of Physiology, University of Tennessee Health Science Center, Memphis, TN, USA

**Keywords:** Autologous stem cell transplantation, CD34+ cells, Poor mobilization, Predictive model

## Abstract

**Introduction:**

Inadequate stem cell mobilization remains a major challenge in autologous stem cell transplantation. Although multiple risk factors for poor mobilization have been suggested, their clinical utility is limited. This study aimed to identify predictors of poor mobilization and develop a clinically applicable predictive model.

**Material and Methods:**

A retrospective analysis of patients who underwent autologous stem cell transplantation was conducted. Poor mobilization was defined as a total CD34^+^ cell collection of less than 2 × 10⁶ cells/kg or the requirement for extended apheresis with multiple collection cycles.

**Results:**

A total of 430 patients with Hodgkin’s lymphoma (n = 134), non-Hodgkin’s lymphoma (n = 113), and multiple myeloma (n = 183) were included in this study. Poor mobilization was observed in 28.6% (123/430) of patients. A predictive model was developed using multivariate analysis, achieving an area under the receiver operating characteristic curve of 0.97 (p-value <0.001) when including the CD34^+^ cell count and 0.98 (p-value <0.001) when excluding it. Independent risk factors for poor mobilization included low platelet count, bone marrow infiltration, advanced disease status, and exposure to ≥2 alkylating agents. The final model demonstrated a specificity of 98.7% (95% CI: 96.7–99.6%) and a sensitivity of 91.1% (95% CI: 84.6–95.4%).

**Conclusion:**

This predictive model can be applied before the initiation of the mobilization process. With sensitivity, specificity, positive predictive value, and positive likelihood ratio all exceeding 90% in both versions, the model demonstrates high effectiveness and clinical utility.

## Introduction

Autologous hematopoietic stem cell transplantation (aHSCT) is a therapeutic intervention for patients with lymphoma and multiple myeloma (MM) [1,2]. The procedure is typically performed using peripheral blood stem cells (PBSCs). Because hematopoietic stem cells (HSCs) normally circulate in the peripheral blood in very low numbers, they must first be mobilized into the circulation before collection by apheresis [3].

Poor mobilization can result in insufficient PBSC yield for aHSCT [4,5]. This represents a significant challenge, as a minimum of 2 × 10⁶ CD34^+^ cells/kg is required to ensure successful transplantation. This threshold is necessary to achieve rapid and sustained hematopoietic recovery, while also reducing the risks of prolonged hospitalization, blood transfusion requirements, and infection [6,7]. The optimal HSC dose is approximately 5 × 10⁶ CD34^+^ cells/kg [8]. Failure to achieve adequate mobilization may necessitate re-mobilization or, in some cases, prevent transplantation altogether.

Several factors have been associated with an increased risk of mobilization failure, including bone marrow infiltration, older age, and myelodysplastic changes [9, 10, 11]. However, the predictive accuracy of these parameters for identifying suboptimal mobilization remains uncertain. Although predictive models have been proposed to address this limitation [12], their applicability across different patient populations has not been fully validated. Early identification of patients at high risk for poor mobilization is essential, as it could minimize resource use, facilitate timely consideration of alternative strategies, and ultimately improve patient outcomes.

This study aimed to identify predictive factors and develop a new risk model for poor mobilization in aHSCT candidates. Additionally, the study validated an existing predictive model [12], developed a novel scoring system based on specific criteria, and compared their performance to optimize clinical decision-making.

## Material and methods

This retrospective study was conducted at the Hematology, Oncology, and Stem Cell Transplantation Research Center, Shariati Hospital in Tehran, Iran. Patients eligible for aHSCT with a diagnosis of MM, non-Hodgkin lymphoma (NHL), or Hodgkin’s disease (HD) between January 2013 and December 2021 were included. The study protocol was approved by the Ethics Committee of Tehran University of Medical Sciences, and all data were collected and analyzed anonymously. Diagnoses of lymphoma were confirmed by pathology review, and diagnoses of MM were established jointly by hematologists and pathologists.

The inclusion criterion was age ≥18 years. Patients with acute or chronic leukemia or those previously mobilized with plerixafor were excluded, as the latter group typically represents poor mobilizers and could bias the predictive analysis. Clinical data, including disease characteristics, prior therapies, mobilization regimen, and apheresis outcomes, were recorded.

Mobilization failure was defined as a total CD34^+^ cell yield of <2 × 10⁶ cells/kg or the requirement for large-volume apheresis involving multiple collection cycles. Stem cell collections were performed using a continuous-flow cell separator following the institutional protocol.

The primary dependent variable was poor mobilization. Independent variables were defined as follows:

• Extensive bone marrow (BM) infiltration was defined based on the most recent BM assessment performed prior to stem cell mobilization, and not at the time of initial diagnosis. Extensive BM infiltration was considered as >60% plasma cells in patients with MM or >30% lymphomatous infiltration in patients with lymphoma.
• Advanced-phase disease: prior treatment with at least two lines of cytotoxic chemotherapy.
• Prior marrow-toxic exposure: previous therapy with lenalidomide, melphalan, or fludarabine.
• Extensive radiotherapy: prior radiation involving marrow-bearing sites.

### Statistical analysis

The association between individual variables and poor mobilization was first assessed using univariate logistic regression, with results expressed as odds ratios (ORs) and 95% confidence intervals (95% CIs). Subsequently, multivariate logistic regression with forward stepwise selection was performed to identify independent predictors of mobilization outcomes.

The GITMO (Gruppo Italiano Trapianto Midollo Osseo) consensus score and the Predicted Poor Mobilizer (pPM) [12] were applied to this dataset generating scores for each patient according to the respective criteria in order to assess the reliability of previously published models. Given the central importance of the pre-apheresis CD34^+^ cell count, this study developed two distinct predictive models: one including the pre-mobilization CD34^+^ cell count and one excluding it. The primary objective was to design a model capable of estimating the probability of poor mobilization at the earliest possible stage, prior to the initiation of apheresis.

Each model classified patients into two categories based on their final score: predicted poor mobilizers and predicted successful mobilizers. Final scores were calculated using different cut-offs, with the cut-off selection ultimately dependent on clinical preference. The objective was to develop a scoring system capable of reliably identifying patients at high risk of mobilization failure; therefore, the threshold that maximized the positive likelihood ratio (LR) was selected. Model performance was assessed by calculating sensitivity, specificity, positive predictive value (PPV), negative predictive value (NPV), and positive and negative LRs, each reported with 95% CIs. LRs and their 95% CIs were expressed as ratios of proportions.

Internal validity of the final model was evaluated through cross-validation. Calibration was assessed using the Hosmer-Lemeshow goodness-of-fit test and by plotting calibration curves comparing observed versus predicted probabilities. Discriminative ability was evaluated using receiver operating characteristic (ROC) curve analysis with the area under the ROC curve (AUC) being calculated according to previously described methods [13]. AUCs were compared using the estimated covariance matrix.

The required sample size was determined based on the primary outcome of poor mobilization. Assuming a 20% poor mobilization rate, a power of 80%, and α = 0.05, detection of an AUC ≥ 0.6 required a total of 405 mobilization procedures (including 81 failures). Statistical analyses were performed using SPSS version 24 (IBM Corp., Armonk, NY) and MedCalc version 20 (MedCalc Software, Ostend, Belgium). A p-value <0.05 was considered statistically significant.

## Results

A total of 430 patients, including Hodgkin’s lymphoma (n = 134), non-Hodgkin’s lymphoma (n = 113), and MM (n = 183), were enrolled in this study. The median age was 47 years. The median number of chemotherapy courses prior to mobilization was nine. Overall, 15.5% of patients had received prior marrow-toxic exposure, including melphalan or fludarabine in lymphoma patients and maintenance lenalidomide in MM patients. At the time of mobilization, 17.5% of patients were in partial remission, while the remainder had achieved complete remission. BM biopsy was performed in all patients prior to mobilization. Median BM infiltration was 35% in patients with mobilization failure compared with 45% in patients with successful mobilization. Baseline demographic and clinical characteristics by disease group are summarized in Table 1, Table 2.

**Table 1:** Qualitative characteristics of patients based on disease type.

		**Diagnosis**									
		**HL** (n = 134)		**NHL** (n = 113)		**MM** (n = 183)			**Total** (n = 430)		
		**Failed mobilization**		**Failed mobilization**		**Failed mobilization**			**Failed mobilization**		
		**Yes**	**No**	**Yes**	**No**		**Yes**	**No**		**Yes**	**No**
		n (%)	n (%)	n (%)	n (%)		n (%)	n (%)		n (%)	n (%)
**Diagnosis**		44	90	37	76		42	141		123	307
Gender	Female	19 (36)	33 (64)	15 (37)	25 (63)		17 (23)	57 (77)		51 (31)	115 (69)
	Male	25 (31)	57 (69)	22 (30)	51 (70)		25 (23)	84 (77)		72 (27)	192 (73)
Extensive BM involvement	No	41 (35)	76 (65)	31 (33)	64 (67)		0 (0)	4 (100)		72 (33)	144 (67)
	Yes	3 (18)	14 (82)	6 (33)	12 (67)		42 (24)	137 (76)		51 (24)	163 (76)
Refractory disease	No	30 (31)	66 (69)	31 (34)	59 (66)		39 (22)	139 (78)		100 (28)	264 (72)
	Yes	14 (37)	24 (63)	6 (26)	17 (74)		3 (60)	2 (40)		23 (35)	43 (65)
Advanced phase disease	No	21 (23)	70 (77)	13 (18)	59 (82)		31 (20)	125 (80)		65 (20)	254 (80)
	Yes	23 (54)	20 (46)	24 (59)	17 (41)		11 (41)	16 (59)		58 (52)	53 (47)
Relapse	No	10 (30)	23 (70)	11 (23)	37 (77)		40 (24)	128 (76)		61 (25)	188 (75)
	Yes	34 (34)	67 (66)	26 (40)	39 (60)		2 (13)	13 (87)		62 (34)	119 (66)
Previous extensive radiotherapy	No	28 (26)	78 (74)	23 (25)	69 (75)		36 (22)	128 (78)		87 (24)	275 (76)
	Yes	16 (57)	12 (43)	14 (67)	7 (33)		6 (32)	13 (68)		36 (53)	32 (47)
Disease status at mobilization	CR	24 (26)	70 (74)	26 (30)	62 (70)		35 (20)	138 (80)		85 (24)	270 (76)
	PR	20 (50)	20 (50)	11 (44)	14 (56)		7 (70)	3 (30)		38 (51)	37 (49)
Large volume apheresis	No	17 (16)	90 (84)	9 (11)	76 (89)		12 (8)	141 (92)		38 (11)	307 (89)
	Yes	27 (100)	0 (0)	28 (100)	0 (0)		30 (100)	0 (0)		85 (100)	0 (0)
Toxic marrow exposure	No	43 (32)	90 (68)	37 (33)	75 (67)		8 (7)	110 (93)		88 (24)	275 (76)
	Yes	1 (100)	0 (0)	0 (0)	1 (100)		34 (52)	31 (48)		35 (52)	32 (48)
Maintenance IMiDs	No	44 (33)	90 (67)	37 (33)	76 (67)		8 (7)	110 (93)		89 (24)	276 (76)
	Yes	0 (0)	0 (0)	0 (0)	0 (0)		34 (52)	31 (48)		34 (52)	31 (48)
≥ 2 Alkylating agents	No	4 (10)	37 (90)	10 (20)	41 (80)		41 (23)	141 (77)		55 (20)	219 (80)
	Yes	40 (43)	53 (57)	27 (44)	35 (56)		1 (100)	0 (0)		68 (44)	88 (56)
Previous alkylating chemotherapy	No	0 (0)	0 (0)	1 (100)	0 (0)		33 (25)	97 (75)		34 (26)	97 (74)
	Yes	44 (33)	90 (67)	36 (32)	76 (68)		9 (17)	44 (83)		89 (30)	210 (70)
Use of IMiDs	No	44 (33)	90 (67)	37 (33)	75 (67)		7 (17)	34 (83)		88 (31)	199 (69)
	Yes	0 (0)	0 (0)	0 (0)	1 (100)		35 (25)	107 (75)		35 (25)	108 (75)

*: percentage in parentheses refer to rows in each category.

BM: bone marrow; HL: Hodgkin disease; NHL: non-Hodgkin lymphoma; MM: multiple myeloma; IMiDs: Lenalidomide or Thalidomide.

**Table 2:** Quantitative characteristics of patients based on disease type.

	**Diagnosis**														
	**HL**			**NHL**					**MM**				**Total**		
	$\tilde{x}$	Min	Max	$\tilde{x}$		Min	Max		$\tilde{x}$	Min	Max		$\tilde{x}$	Min	Max
Age (years)	34.21	17.90	67.55	43.88		17.70	64.88		55.32	28.87	77.03		46.96	17.70	77.03
BM infiltration (%)	40	20	80	40		25	70		40	20	85		40	20	85
Harvested volume (mL)	540	180	2100	559		180	1950		418	164	2685		470	164	2685
Harvested nucleated cell (x10^7^ /kg)	13.55	3.60	68.80	13.36		3.90	44.65		13.01	1.74	31.24		13.25	1.74	68.80
pre-mobilization CD34^+^ count (x10^6^ /kg)	15.10	1.00	70.00	15.00		2.50	75.00		18.00	1.00	40.00		17.00	1.00	75.00
Harvested CD34^+^ (x10^6^ /kg)	3.00	.50	10.20	2.80		.52	12.00		3.40	.46	16.04		3.12	.46	16.04
WBC (/mm^3^)	5.05	2.00	12.40	4.70		1.50	11.30		5.70	1.70	22.40		5.10	1.50	22.40
Hemoglobin (mg/dL)	12.10	7.70	17.60	12.30		7.80	17.50		12.80	9.40	18.70		12.50	7.70	18.70
Platelet (/mm^3^)	160	22	414	162		34	419		193	31	387		175	22	419
Number of chemotherapy courses	12	6	26	11		6	16		6	4	16		9	6	26

BM: bone marrow; HL: Hodgkin disease; NHL: non-Hodgkin lymphoma; MM: multiple myeloma;

$\tilde{x}:$median.

Overall, 123 patients (28.6%) were classified as poor mobilizers, while 307 patients (71.4%) achieved adequate stem cell collection for transplantation. The causes of suboptimal mobilization were an unsatisfactory peak CD34^+^ cell count (<20 cells/µL) in 19 (15.4%) patients, insufficient total CD34^+^ yield (≤2 × 10⁶ cells/kg) in ten (8.1%) patients, and both criteria in 94 (76.4%).

Patients with failed mobilization demonstrated significantly greater BM infiltration and lower pre-mobilization CD34^+^ cell counts compared with those who mobilized successfully. Quantitative parameters associated with mobilization outcome are detailed in Table 3.

**Table 3:** Quantitative parameters based on failed mobilization.

	**Failed mobilization**						
	**No**				**Yes**		
	$\tilde{x}$	**Min**	**Max**		$\tilde{x}$	**Min**	**Max**
Age (years)	46.90	18.28	69.35		46.96	17.70	77.03
Bone marrow infiltration (%)	45	25	85		35	20	55
Harvested volume (mL)	439	164	2685		600	250	2100
Harvested nucleated cell (x10^7^ /kg)	13.05	1.74	68.80		13.63	3.60	52.07
Pre-mobilization CD34^+^ (x10^6^ /kg)	20.00	3.00	75.00		8.00	1.00	25.00
Harvested CD34^+^ (x10^6^ /kg)	3.90	2.00	16.04		1.70	.46	8.80
WBC count (/mm^3^)	5.50	1.60	22.40		4.20	1.50	12.70
Hemoglobin (mg/dL)	13.00	8.10	18.70		11.60	7.70	15.40
Platelet count (/mm^3^)	200	31	419		110	22	322
Number of chemotherapy courses	9	6	18		10	8	26

### Poor mobilization risk factors

Univariate regression analysis was used to evaluate the association between different variables and poor mobilization. Significant predictors (p-value <0.001) included intact BM reserve, history of relapse, prior extensive radiotherapy, advanced disease status at mobilization, BM infiltration, pre-mobilization CD34^+^ cell count, white blood cell count, hemoglobin level, platelet count, number of prior chemotherapy courses, toxic marrow exposure (fludarabine, melphalan, or lenalidomide), and treatment with more than two alkylating agents. In contrast, gender, age, refractoriness, and underlying hematologic diagnosis were not significantly associated with mobilization failure (Table 4).

**Table 4:** Mobilization failure predictors according to univariate logistic regression.

**Candidate factor**	**β**	**p-value**	**OR**	**95% CI**	
				**Lower**	**Upper**
Gender (Female)	0.173	0.427	1.189	0.776	1.821
Age	−0.003	0.744	0.997	0.982	1.013
Diagnosis		0.089			
HL	0.495	0.051	1.641	0.997	2.702
NHL	0.478	0.073	1.613	0.957	2.719
Intact bone marrow	−0.462	0.032	0.630	0.413	.962
Refractory disease	0.349	0.219	1.417	0.813	2.472
Advanced phase disease	1.457	<0.0001	4.293	2.706	6.810
Relapse	0.479	0.026	1.614	1.059	2.460
Previous extensive radiotherapy	1.272	<0.0001	3.569	2.093	6.086
Disease status at mobilization (PR)	1.186	<0.0001	3.274	1.958	5.475
BM infiltration	−0.175	<0.0001	0.840	0.809	0.871
Pre CD34^+^	−0.572	<0.0001	0.564	0.496	0.642
WBC count	−0.290	<0.0001	0.748	0.660	0.848
Hemoglobin level	−0.621	<0.0001	0.537	0.455	0.635
Platelet count	−0.053	<0.0001	0.949	0.938	0.959
Number of chemotherapy courses	0.150	<0.0001	1.162	1.091	1.237
Toxic marrow exposure	1.233	<0.0001	3.430	2.007	5.864
>2 Alkylating agents	1.128	<0.0001	3.091	2.005	4.766

HL: Hodgkin lymphoma; NHL: Non-Hodgkin lymphoma; PR: partial remission; OR: odds ratio; 95% OI: 95% confidence interval.

### Verifying previous models

This dataset was first applied to the previously proposed pPM and GITMO scoring systems [20]. In the pPM model, scores ranged from 8.7 to 24.7 with a median of 13.5. Using the published cut-off of 7.86, all patients in the cohort were classified as poor mobilizers, indicating limited discriminative value in this setting.

For the GITMO score, values ranged from 0–7 with a median of 1. Based on the consensus, the cut-off was set at ≥2. At this threshold, sensitivity for predicting mobilization failure was 70.7% (95% CI: 61.8–78.6%) and specificity was 69.1% (95% CI: 63.6–74.2%). The positive likelihood ratio was 2.3 (95% CI: 1.9–2.8). With a poor mobilization prevalence of 28.6% in this cohort, the positive predictive value (PPV) was 47.8% (95% CI: 42.8–52.8%) and the AUC for predicting poor mobilization was 0.70 (95% CI: 0.66–0.75).

### Modeling

Given the modest performance of the pPM and GITMO scores in the current dataset, further analyses were conducted to develop a more robust predictive model. Adjusting cut-off values within these models resulted in reduced sensitivity and specificity with positive predictive values (PPVs) ranging only from 40.3% to 56.7%.

The predictive role of the CD34^+^ cell count in the mobilization outcome is well established [4, 5, 6, 7, 8]. In accordance with the primary objective of this study to develop a model capable of identifying poor mobilizers prior to apheresis, two distinct models were constructed: one including the pre-mobilization CD34^+^ cell count and one excluding this variable.

Multivariate logistic regression was performed using the variables found significant in the univariate analysis. In the model excluding the CD34^+^ cell count, the final regression model was formulated after five steps using forward stepwise selection. The improvement employing the chi-square test was 344.64 (p-value <0.001), indicating strong statistical significance. The Cox-Snell and Nagelkerke R² values demonstrated that the model explained between 55% and 79% of the variance in poor mobilization. This model achieved a sensitivity of 89.4%, specificity of 94.1%, PPV of 89.4%, NPV of 94.1%, and an overall accuracy of 92.8%.

When the CD34^+^ cell count was incorporated into the multivariate regression, the final model was achieved after four steps. The improvement employing the chi-square test was 415.28 (p-value <0.001), with Cox–Snell and Nagelkerke R² values showing that 62% to 89% of the variance in outcome could be explained.

Independent predictors identified in the two models were as follows:

− Model excluding the pre-mobilization CD34^+^ cell count: platelet count, BM infiltration, advanced disease status, full-course chemotherapy, and the use of more than two alkylating agents.
− Model including the pre-mobilization CD34^+^ cell count: platelet count, BM infiltration, extensive radiotherapy, and the CD34^+^ cell count.

**Figure 1:**
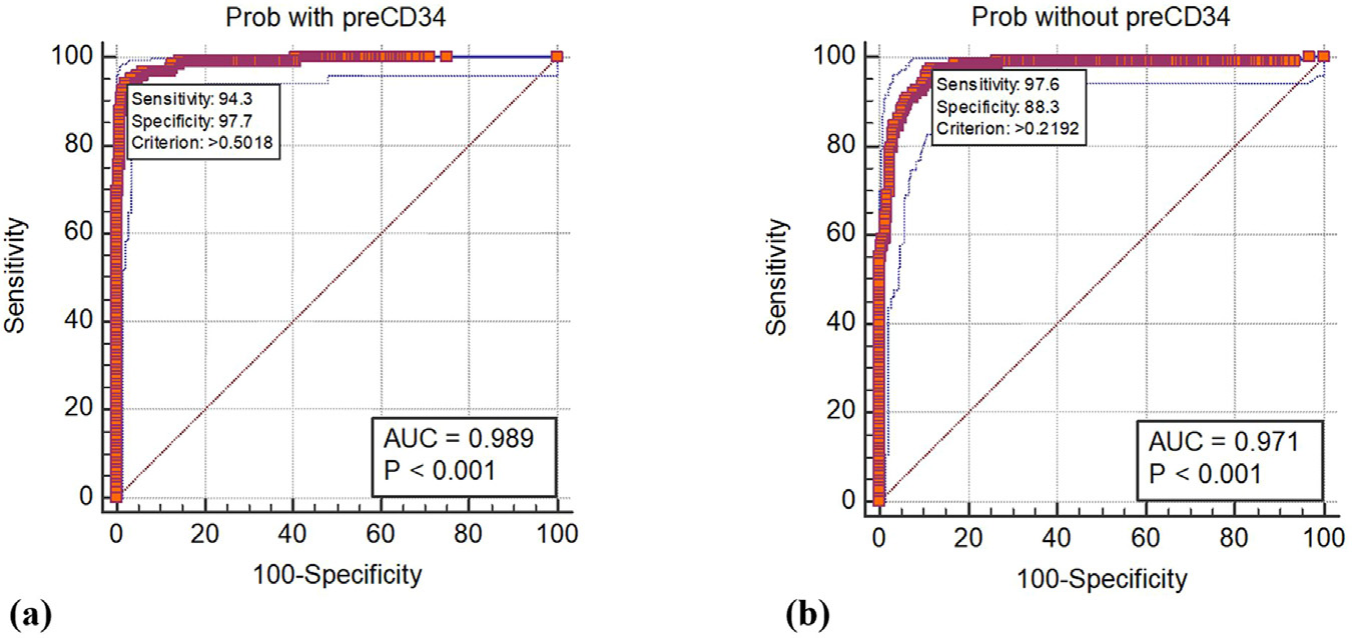
Area under the receiver operating characteristic curves for poor mobilization using (**a**) the model including the pre-mobilization CD34^+^ count and (**b**) the model excluding the pre-mobilization CD34^+^ count.

Cut-off optimization yielded excellent discriminative performance. In the CD34^+^ model, a threshold >0.219 produced an AUC of 0.97, with sensitivity 97.6% and specificity 88.3%. In the model excluding the CD34^+^ count, a cut-off >0.502 resulted in an AUC of 0.98, with sensitivity 94.3% and specificity 97.7% (Figure 1; Table 5, Table 6).

**Table 5:** Statistical measures of predicting models for poor mobilization.

Measure	Value	95% CI
Model excluding the pre-mobilization CD34^+^ cell count		
Cut-off	0.71	
Sensitivity	0.911	0.846 - 0.954
Specificity	0.987	0.967 - 0.996
Positive Likelihood Ratio	69.89	26.36 −185.31
Negative Likelihood Ratio	0.09	0.05 - 0.16
Disease prevalence*	28.60%	24.38% - 33.13%
Positive Predictive Value*	0.965	0.913 - 0.987
Negative Predictive Value*	0.965	0.940 - 0.979
Accuracy*	0.965	0.943 - 0.980

Model including the pre-mobilization CD34^+^ cell count		

Cut-off	0.43	
Sensitivity	0.911	0.846 - 0.954
Specificity	0.935	0.901 - 0.959
Positive Likelihood Ratio	13.98	9.12 - 21.43
Negative Likelihood Ratio	0.10	0.05 - 0.17
Disease prevalence*	28.60%	24.38% - 33.13%
Positive Predictive Value*	0.848	0.785 - 0.896
Negative Predictive Value*	0.963	0.937 - 0.978
Accuracy*	0.928	0.899 - 0.950

95% CI: 95% confidence interval.

⁎ These values are dependent on the disease prevalence.

**Table 6:** Independent predictive factors for mobilization failure identified by forward stepwise method with multiple logistic regression.

**Model excluding the pre-mobilization CD34^+^ cell count**					
**Variables in the Equation**	**β**	**p-value**	**Odds ratio**	**95% CI**	
				**Lower**	**Upper**
					
Advanced phase disease	1.086	0.010	2.961	1.292	6.788
Bone marrow infiltration	−0.156	<0.0001	0.855	0.810	0.904
Platelet count	−0.043	<0.0001	0.958	0.946	0.970
Full courses of previous therapy	2.443	0.001	11.512	3.660	36.204
More than two Alkylating	1.615	0.001	5.027	2.000	12.637


**Model including the pre-mobilization CD34^+^ cell count**					
**Variables in the Equation**	**β**	**p-value**	**Odds ratio**	**95% CI**	
				**Lower**	**Upper**
					
Previous extensive radiotherapy	1.933	0.012	6.912	1.525	31.333
Bone marrow infiltration	−0.118	0.001	0.889	0.830	0.952
Pre-mobilization CD34^+^ cell count	−0.518	<0.0001	0.596	0.502	0.708
Platelet count	−0.033	<0.0001	0.967	0.954	0.981

95% CI: 95% confidence interval.

The probability of mobilization failure according to the model score without the CD34^+^ cell count, can be calculated as below:

$$\text{Poor}\;\text{Mobilization}\;\text{Probability}\;=\;\frac{e^{\left(model\;score-10.082\right)}}{e^{\left(model\;score-10.082\right)\;}+1}$$

**Figure 2:**
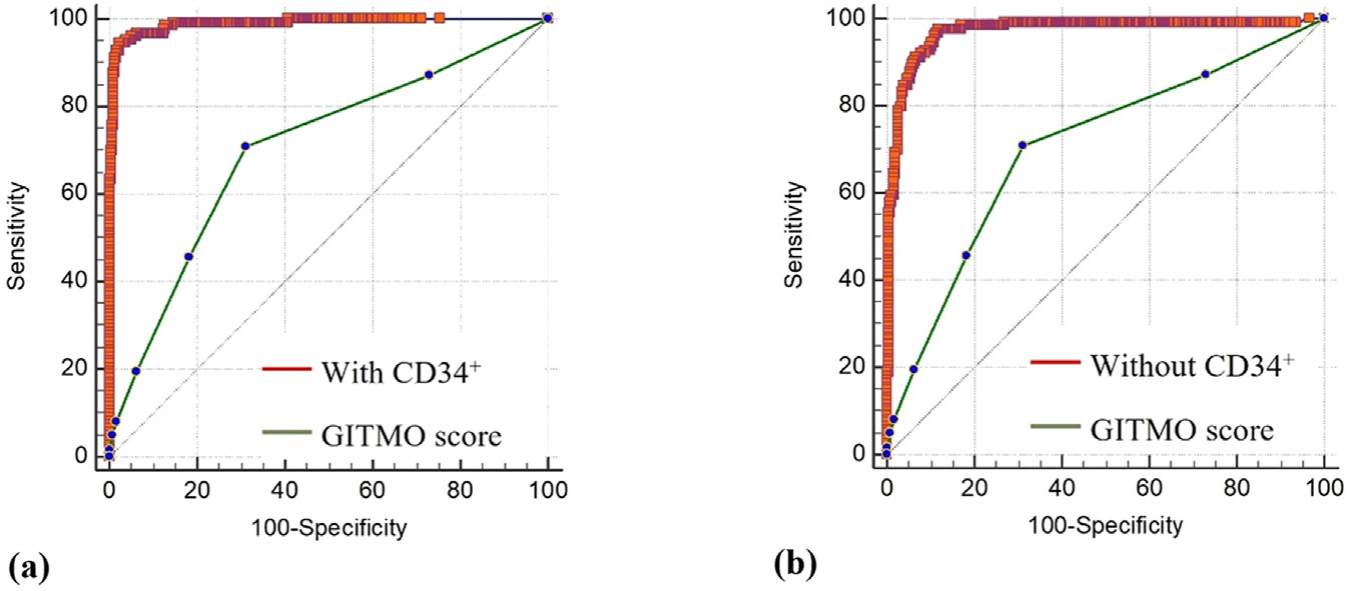
Comparing the area under the receiver operating characteristic curve of the GITMO (Gruppo Italiano Trapianto Midollo Osseo) score with (**a**) the model including the pre-mobilization CD34^+^ count and (**b**) the model excluding the pre-mobilization CD34^+^ count.

The AUCs of the GITMO score, pPM score and the scores of the models with and without CD34^+^ inclusion were compared. The AUC of previous models were significantly inferior to both the models constructed in this study (p-value <0.0001). Figure 2 shows the result.

## Discussion

This study evaluated mobilization outcomes in patients undergoing aHSCT, with the primary aim of identifying prognostic risk factors and assessing the applicability of previously published predictive scores (GITMO and pPM). A secondary objective was to develop a practical predictive model capable of identifying patients at high risk of mobilization failure, ideally independent of the well-established predictor: pre-apheresis CD34^+^ cell count. To this end, four models were examined: the GITMO score, the pPM score, and two newly developed models (one including and one excluding the pre-mobilization CD34^+^ cell count).

The pPM score demonstrated poor performance in the current dataset, essentially failing to predict poor mobilization. The GITMO score also showed limited accuracy, with modest sensitivity (70%), specificity (69%), and an AUC of 0.70. In contrast, the new predictive models performed substantially better, each achieving an AUC greater than 0.9, with excellent sensitivity, specificity, and PPVs. Using optimized cut-offs of 0.71 (model excluding the pre-mobilization CD34^+^ cell count) and 0.43 (model including the pre-mobilization CD34^+^ cell count), both models demonstrated strong clinical utility in predicting mobilization failure.

Several factors may explain the discrepancy between the present findings and those of prior studies. The pPM score developed by Olivieri et al. [14], was based on variables markedly different from those of this patient population. For example, in this study the median number of prior chemotherapy courses was nine (range: 2–26), compared with only 1–6 in the Italian study. Likewise, conditioning regimens differed substantially: Olivieri et al. identified granulocyte colony-stimulating factor (G-CSF) use without chemotherapy as a strong negative predictor (β = 2.25), whereas in this study all patients received G-CSF alone. Age distribution also varied, with the present median patient age being 46 years, while 26% of patients in the Italian cohort were younger than 45 years. Moreover, Italian patients tended to undergo aHSCT earlier in their disease course, resulting in lower cumulative exposure to marrow-toxic therapy compared to this study.

These contextual differences highlight why previously published models may lack external validity across diverse patient populations. Despite recent advances in peripheral blood stem cell collection including apheresis instrumentation, mobilization failure remains a challenge, with reported rates ranging from 5% to 40% [15, 16, 17]. The transfused stem cell dose, directly linked to the number of CD34^+^ cells collected, is a key determinant of engraftment success [14,18]. While circulating CD34^+^ cell counts remain the most reliable predictor of mobilization success, their utility is limited by the fact that they are only available immediately prior to apheresis. This delay prevents early identification and stratification of patients who might benefit from alternative mobilization strategies.

The new models address this limitation by incorporating additional predictive variables that are assessable before apheresis. In the model excluding the CD34^+^ cell count, independent predictors of mobilization failure included platelet count, BM infiltration, advanced disease status, history of full-course chemotherapy, and prior exposure to more than two alkylating agents. When the CD34^+^ cell count was included, the independent predictors were platelet count, BM infiltration, pre-mobilization CD34^+^ cell count, and prior extensive radiotherapy. These findings suggest that predictive models can be tailored to different clinical contexts, either allowing earlier patient stratification without pre-mobilization CD34^+^ cell data or achieving maximum predictive accuracy including the pre-mobilization CD34^+^ count.

A 28.6% rate of mobilization failure was observed in older patients in this study, whereas other investigators such as Rossi et al. [19] and Wuchter et al. [20] reported rates as high as 46%. This discrepancy is likely attributable to differences in mobilization techniques between studies, but it warrants further investigation. Demiroğlu et al. reported that higher pre-mobilization platelet counts, and the use of chemotherapy-based mobilization regimens were associated with greater mobilization success. While the platelet count was also identified as a significant predictor in the present study, the mobilization regimen could not be examined since our protocol was chemotherapy-free [21].

Although no single measure of accuracy can fully capture the clinical value of a predictive tool, the AUC is widely considered a reliable indicator of discriminative performance, as it is independent of cut-off values or disease prevalence [22]. In this respect, the new models demonstrated excellent predictive capability, each achieving an AUC greater than 0.95.

One important consideration in evaluating predictive scores across studies is the discrepancy between the minimal threshold and the optimal target dose of CD34^+^ cells. Defining poor mobilization solely by the minimum cut-off of 2 × 10⁶ CD34^+^ cells/kg may underestimate clinical requirements, as higher doses are often needed depending on the patient’s condition. This variability may affect the accuracy and applicability of predictive models.

The primary goal of this study was to establish a practical tool for decision-making before mobilization. These new models incorporate easily obtainable variables available prior to apheresis and classify patients as ‘poor mobilizers’ or ‘successful mobilizers.’ Such information can guide clinical planning, including reconsideration of mobilization targets, early use of CXCR4 antagonists, cytokine therapy, or large-volume apheresis. The strong positive likelihood ratio (>10), combined with sensitivity and specificity above 85%, supports the reliability and potential generalizability of these models across different settings.

In the present cohort, plerixafor was used only on-demand, limiting the ability to evaluate or recommend its upfront use. Nevertheless, based on this model, it would be reasonable to consider early administration of plerixafor in patients classified as poor mobilizers, in line with recent reports [36]. Similarly, because G-CSF–based mobilization was employed exclusively, conclusions regarding chemotherapy-based mobilization strategies cannot be provided.

In addition to cytokine-based approaches, chemotherapy-based mobilization strategies, particularly vinorelbine combined with G-CSF, have been reported as effective alternatives for poor mobilizers, leading to higher pre-apheresis CD34⁺ cell counts, predictable timing of collection (typically on days 7–8), and acceptable toxicity with a relatively low risk of infectious complications, while remaining substantially more cost-effective than plerixafor [4,6,10].

As a future direction, prospective studies could apply this predictive framework prior to mobilization, without reliance on circulating CD34⁺ counts, to identify poor mobilizers and randomize them between G-CSF–based mobilization with early or on-demand plerixafor versus chemotherapy-based mobilization with vinorelbine. In addition to apheresis success rates, such studies should incorporate pharmacoeconomic analyses to assess the cost-effectiveness of these competing strategies.

Finally, although the findings of this study suggest that adapting mobilization approaches according to the predictive model may be clinically beneficial, external validation in an independent cohort is required. Furthermore, prospective clinical trials are essential to confirm the effectiveness and safety of implementing this model in routine practice.

## Conclusion

This study developed a predictive model for inadequate mobilization that excludes the pre-mobilization CD34^+^ cell count, making it applicable prior to the initiation of the mobilization process. Both versions of the model demonstrated sensitivity, specificity, positive predictive value, and positive likelihood ratio exceeding 90%. Early identification of poor mobilizers enables individualized monitoring, supports optimized decision-making, and ultimately enhances patient outcomes.

## Funding

This research received no specific grant from funding agencies in the public, commercial, or non-profit sectors.

## Data availability

The data that support the findings of this study are available from the corresponding author upon reasonable request.

## Conflicts of interest

The authors declare no conflicts of interest.
